# Bad Taste Protects Fruit Flies from Eating a Toxic Amino Acid in Plants

**DOI:** 10.1371/journal.pbio.1000128

**Published:** 2009-06-30

**Authors:** Robin Meadows

**Affiliations:** Freelance Science Writer, Fairfield, California, United States of America

## Abstract

An orphan G-protein-coupled gustatory receptor mediates detection of the plant poison L-canavanine in fruit flies.

**Figure pbio-1000128-g001:**
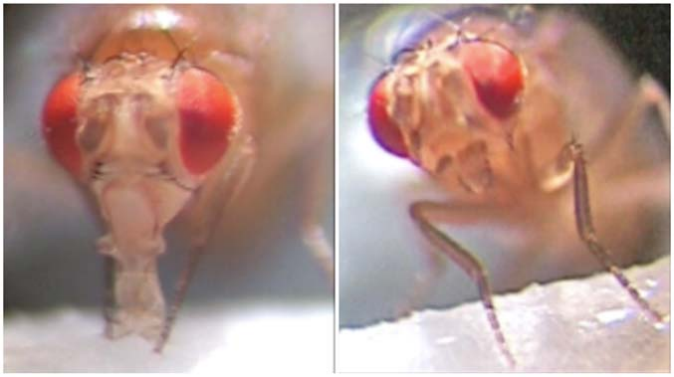
Fruit flies will drink a solution if it is considered attractive, like a sugar solution (left). However, when L-canavanine is added to the sugar solution, fruit flies retract their proboscises, avoiding ingesting this plant toxin (right).

[Fig pbio-1000128-g001]Besides enhancing the pleasure of eating, our sense of taste can steer us away from poisonous foods. Many plants, for example, produce bitter-tasting toxins, such as caffeine and quinine, to deter predation by herbivores from cows to insects. But it remains a mystery how animals developed their capacity to detect—and so avoid—the tens of thousands of plant toxins, which include alkaloids, phenolics, and nonprotein amino acids. The best-known toxic nonprotein amino acid is L-canavanine, which accumulates in the seeds of alfalfa (*Medicago sativa*) and many other leguminosae. L-canavanine is so similar to the amino acid L-arginine that it gets incorporated into proteins, rendering them dysfunctional. A few insects have developed strategies for counteracting this toxin, including larvae of the beetle *Caryedes brasiliensis*, which depend entirely on seeds of the legume *Dioclea megacarpa* and catabolize L-canavanine into harmless compounds. However, the vast majority of insects are susceptible to L-canavanine, and little is known about their ability to detect it in plants.

In this issue of *PLoS Biology*, new research by Yves Grau and colleagues shows that the fruit fly *Drosophila melanogaster* can taste L-canavanine. G protein-coupled receptors (GPCRs) are seven-transmembrane proteins that sense molecules outside of a cell and then activate a signaling pathway inside that triggers the cell's response to the molecules. In mammals and insects, GPCRs include metabotropic glutamate receptors (mGluRs) that are activated by glutamate, a neurotransmitter amino acid. The researchers had previously shown that *Drosophila* have an mGluR-derived receptor found only in insects, called DmXR, which differs enough from mGluR that it is not activated by glutamate but similar enough that it could still be activated by a molecule having amino acid properties.

Now, Grau and colleagues report that *Drosophila* rely on DmXR to taste the plant toxicant L-canavanine. This comes as a surprise, because all of the other known insect taste receptors belong to a family of gustatory receptors called Grs, which, in fruit flies, includes the caffeine receptor Gr66a. Grs are seven-transmembrane proteins that are distantly related to the GPCRs family. The researchers tested DmXR for L-canavanine sensitivity, because some other members of the fly family eat seeds, which can be rich in this toxic amino acid. In contrast, *Drosophila* eat decaying organic matter, landing on it to taste it with their legs, which have taste neurons that contain receptors for sugars, water, and bitter compounds.

To see if L-canavanine activates the DmX receptor, Grau and colleagues expressed the receptor in human embryonic kidney (HEK) cells and treated them with either L-canavanine or the similar amino acid L-arginine. They found that the former activated the receptor while the latter did not. To see if DmXR binds L-canavanine, the researchers expressed a mutant of this receptor with an altered binding site in HEK cells and treated them with L-canavanine. They found that this toxic amino acid failed to activate the mutant DmXR.

The next steps involved confirming that *Drosophila* are susceptible to and avoid L-canavanine. Grau and colleagues found that while fruit flies that ate this toxicant suffered no dramatic effects themselves, all of their offspring died as larvae. Taste-tests then showed that *Drosophila* avoid eating L-canavanine. For example, by giving the choice between two colored sugar solutions, while fruit flies usually prefer blue sugar solutions over red ones, they overwhelmingly chose the red when the blue was spiked with L-canavanine. But *Drosophila* mutants that lack the DmXR gene, called *mangetout* (in reference to the snow peas leguminosae, also called “mangetout,” which is French for “eat the whole thing,” including the pod), still vastly preferred the blue sugar solution even when it was spiked with L-canavanine.

Finally, Grau and colleagues showed that DmXR is a gustatory receptor for L-canavanine, based on a combination of behavioral, genetic, and pharmacological evidence. When fruit flies' legs taste a sugar solution, they usually extend their proboscis to drink. But bitter compounds make them more likely to block proboscis extension or to prematurely retract their proboscises. Accordingly, the researchers found that proboscis retraction increased after applying an L-canavanine–spiked sugar solution on the insects' legs. In contrast, this repulsive phenotype was not observed anymore on *mangetout* (*mtt*) mutants.

Another line of evidence that DmXR is a taste receptor acting in bitter-sensitive taste neurons came from preventing *mtt* expression with interference RNA (RNAi), double-stranded RNA that knocks out gene function by degrading the associated messenger RNA. The researchers found that RNAi knockdown of *mtt* in Gr66a taste neurons—which contain caffeine receptors—reduces proboscis retraction in flies on L-canavanine–spiked sugar solutions.

Wrapping up their multi-pronged approach, Grau and colleagues strengthened the case that DmXR is a taste receptor for L-canavanine by showing that it does not regulate synaptic transmission in Gr66a taste neurons. The evidence included that *mtt* mutants do not affect caffeine sensitivity, indicating that DmXR is specific to L-canavanine and does not modify Gr66a activation more generally.

The structural relationship between L-canavanine and caffeine receptors remains to be teased apart. Grau and colleagues raise several possibilities, including that the two receptors are coupled to separate transduction pathways or that, like some *Drosophila* olfactory receptors, Grs are also membrane channels themselves.

The binding site of the DmX taste receptor differs from that of mGluR—its parent glutamate-activated receptor—by just two residues, which interact with the γ-carboxylic group of the neurotransmitter glutamate. This suggests that this mGlu receptor was more suited to evolving into a new taste receptor for L-canavanine than those in the Gr family of previously known *Drosophila* taste receptors. Likewise, Grau and colleagues propose that other taste receptors may also have arisen from outside this family. As insecticide resistance grows, understanding such insect-specific receptors is key to finding new ways of controlling insect pests and the diseases they can carry.

**Mitri C, Soustelle L, Framery B, Bockaert J, Parmentier M-L, et al. (2009) Plant insecticide L-canavanine repels *Drosophila* via the insect orphan GPCR DmX. doi:10.1371/journal.pbio.1000147**

